# Perceived loneliness and the role of cultural and intergenerational belonging: the case of Portuguese first-generation immigrants in Luxembourg

**DOI:** 10.1007/s10433-021-00617-7

**Published:** 2021-04-09

**Authors:** Isabelle Albert

**Affiliations:** Faculty of Humanities, Education and Social Sciences, University of Luxembourg, Esch/Alzette, Luxembourg

**Keywords:** Migration, Luxembourg, Portuguese, Loneliness, Belonging, First-generation

## Abstract

The risk of loneliness for migrants, particularly in older age, has been documented across multiple studies. Migration is a life-changing transition. While often retaining links to their country of origin, an important developmental task for migrants is the establishment of bonds in the receiving country. Drawing on recent studies, I will explore the role of cultural and intergenerational belonging in order to identify both protective and risk factors regarding loneliness in middle and older age in a sample of first-generation immigrants from Portugal living in Luxembourg. The sample comprises *N* = 131 participants (51.9% female) between the ages of 41 and 80 (*M* = 56.08; SD = 7.80) who have on average spent *M* = 31.71 years (SD = 8.81) in Luxembourg and raised children in Luxembourg. They took part in the IRMA project (‘Intergenerational Relations in the Light of Migration and Ageing’) which was funded by the Fonds National de la Recherche Luxembourg. A standardised questionnaire assessed socio-demographic data, aspects of cultural belonging (i.e. cultural attachment to both countries, bicultural identity orientation, acculturative stress), intergenerational belonging (i.e. family cohesion, family conflict, perceived intergenerational value consensus) and perceived loneliness. Results showed that while cultural and intergenerational belonging were protective factors, the strongest predictors for participants’ perceived loneliness were cultural identity conflict and, even more so, intergenerational conflict. Our findings suggest that establishing roots and bonds in the host country is a protective factor against loneliness, whereas the feeling of not fitting in is a strong risk factor.

Daughter: ‘Sure, they were used to seeing only red roofs, it is like this in Portugal.’ Mother: ‘But here the roofs were all black, I would have taken the train and gone back to Portugal immediately, I had left behind such a beautiful country and I didn't like this place.’ Interviewer: ‘And do you still think that about Luxembourg?’ Mother: ‘No, it's much nicer now!’ (laughter)^1^

Migration is a transition in life that entails changes in cultural, social and emotional belonging. Establishing new roots and ties in the receiving country is one of the main tasks of immigrants in their process of socio-cultural and psychological adaptation (Berry 2017). A failure to establish bonds with the receiving country can lead to social isolation and loneliness (Hurtado-de-Mendoza et al. 2014).

Immigrants leave behind their familiar surroundings and have to reorient themselves in a new context. In the new country, they are not only confronted with an unfamiliar physical environment but also with new cultural aspects, such as the values and practices of the host population. A study with immigrants in Canada highlighted the importance of immigrants’ familiarity with cultural aspects in the receiving country for their well-being: immigrants who had a similar language and culture were not lonelier than host nationals in contrast to those from more culturally distant countries and with a different mother tongue (De Gierveld et al. 2015). Furthermore, in a study by Wu and Penning (2015), length of stay reduced perceived loneliness of ageing first-generation immigrants, although immigrant status had a less important effect in the oldest age group where unpredictable age-related events and losses might be more relevant than migrant status itself.

A widely used definition of the experience of loneliness stems from Perlman and Peplau (1984) and refers to loneliness as an individual’s perceived divergence between their desired and their actual social network. Notably, whereas social isolation refers to the objective description of a person’s social contacts, loneliness remains a highly subjective experience. One might feel lonely in spite of a large social network and a high quantity of social contacts, and vice versa, one might have only few social contacts but not feel lonely (Cacioppo et al. 2015).

While loneliness and social isolation are not bound to specific groups and ages, migration can be a risk factor as recent studies suggest (Burholt et al. 2020; Fokkema and Naderi 2013; Victor et al. 2012). As migrants leave relatives and friends behind in their country of origin, they might have a smaller and less satisfying local social network in the receiving country (Wu and Penning 2015). In their representative Canadian sample of adults (aged 60+), Wu and Penning (2015) found more loneliness for first-generation immigrants compared to native Canadians and to second- or third-generation. In a similar vein, a study in the Netherlands showed higher rates of social and emotional loneliness in migrants compared to Dutch participants over the age of 40. These differences were explained by migrants’ lower satisfaction with social relations (Kate et al. 2020).

In addition, older age has often been associated with a higher proneness for loneliness due to a loss of social network partners, reduced social participation, higher probability of health issues and reduced mobility (Beaumont 2013; Kemperman et al. 2019). With increasing age, migrants thus have a double risk for loneliness and social isolation, both due to age-related losses and to missing bonds and resources related to their migration experience.

The present study focusses on loneliness of adults (40+) with a migrant background, namely first-generation Portuguese immigrants to Luxembourg, with the aim of examining cultural and intergenerational belonging as protective factors against loneliness and identifying in how far the feeling of not fitting in, namely the experience of cultural and intergenerational conflict, might place middle-aged and older immigrants at an elevated risk for loneliness. The study will thus provide further insights into the interplay of aspects of cultural and intergenerational belonging for perceived loneliness in the context of migration.

## The sense of belonging and perceived loneliness in the context of migration

Recent studies have focussed on the link between immigrants’ sense of belonging and their experience of loneliness. The sense of belonging is a multifaceted concept, which is related to identification, connectedness, attachment, feeling at home or the feeling of fitting in (Halse 2018). According to Baumeister and Leary (1995), the need to belong is universal. Their definition includes two criteria: the individuals’ need for (positive, not conflict-laden) social interactions and their need for an affective, reliable and caring bond with others. Belonging can refer to different entities, such as place, i.e. the feeling of being attached to geographic areas or symbolic spaces (Antonsich 2010), and social entities, i.e. family or peers, social institutions (e.g. school), or transnational and global networks (Halse 2018). According to Juang et al. (2018), who combine geographical and social aspects of belonging, it is beneficial for immigrants’ adjustment to build up both geographic bonds (i.e. to the place where one lives) and close social ties that make them feel safe and comfortable in the receiving context.

The present study investigates how aspects of belonging on two different levels, namely cultural and intergenerational belonging, are of importance in understanding middle-aged and older immigrants’ loneliness, and these aspects will be outlined in the next sections.

### Cultural belonging

Acculturative processes are key when it comes to the establishment of a sense of belonging to a new living context. While earlier theories conceptualised acculturation as a one-dimensional process, two- or multidimensional models claim that migrants establish new bonds to the receiving country while also retaining ties to the culture of origin (see Van de Vijver 2015). While a new sense of belonging is created, earlier experiences of belonging might leave a lasting imprint (Gamsakhurdia 2018). For instance, in a sample of Turkish and Moroccan migrants living in the Netherlands, Klok et al. (2017) explored the question of how aspects of belonging might be protective factors to reduce the experience of loneliness of migrants. Drawing on Berry’s (2017) acculturation model, they identified five different patterns of belonging based on latent class analysis: marginalised, marginalised/separated, integrated, assimilated and separated migrants. According to their results, marginalised and marginalised/separated migrants felt lonelier than did the migrants in all other clusters. Interestingly, there was no advantage of bicultural belonging over monocultural belonging (integration vs. assimilation or separation clusters). This means that some form of belonging in the receiving country is important, yet the group to which migrants feel they belong is less important (i.e. either to members of the receiving culture or peers from the culture of origin).

When the processes of psychological or socio-cultural adaptation are experienced as difficult to attain or not in line with one’s own cultural expectations, acculturative stress might occur. The concept of acculturative stress describes difficulties of immigrants regarding their intercultural contacts in the receiving society and was coined by Berry (2006, p. 296) as ‘a stress reaction to life events that are rooted in the experience of acculturation’. Acculturative stress is thus linked to the feeling of not belonging and not fitting in and is related to uncertainty, anxiety and depression in the context of migration (Berry 2006).

While acculturation refers to changes in cultural patterns regarding everyday practices, attitudes and beliefs, the ways in which different cultural elements are negotiated within a person’s self-construal have been described by the concept of (multi)cultural identity (Berry 2003). This is important when considering how people actually feel and think about their different cultural belongings. Benet-Martínez and colleagues (e.g. Haritatos and Benet-Martínez 2002) proposed two dimensions to describe how individuals cognitively and affectively integrate different cultural aspects in their identities: (1) compartmentalisation versus blendedness and (2) conflict versus harmony. The former dimension refers to migrants’ either maintaining both (or multiple) cultures they belong to as distinct or experiencing them as overlapping, whereas the latter dimension refers to the experience of tension versus compatibility between their cultures. Furthermore, cultural frame switching was introduced as the behavioural component of bicultural identity, referring to the ability to act competently in different cultural settings and to alternate between them, i.e. behaving according to the respective cultural environment (Benet-Martinez et al. 2002; Hong et al. 2000; LaFromboise et al. 1993; Phinney and Devich-Navarro 1997). While feeling as if one belongs to both cultures can be a resource, having an inner conflict about where to belong can have a negative effect on subjective well-being (Barros and Albert 2020).

With regard to cultural belonging, it is therefore hypothesised that cultural attachment to Luxembourg (LU) and Portugal (PT) as well as the feeling that both cultures are compatible and that one can competently navigate between the two will protect against perceived loneliness, whereas acculturative stress and the feeling that both cultures are conflicting will increase perceived loneliness.

### Intergenerational belonging

Strong family bonds might evolve particularly in the context of migration as family can function as a protection against adversities and as a main source of support (Schönpflug and Bilz 2009). Several studies have reported a higher exchange of support from adult children to their parents and high family obligations in migrant families (Albert et al. 2013; Bordone and de Valk 2016; Merz et al. 2009). Migrant parents have higher expectations of support from their adult children, and their reasons for remaining in the receiving country after retirement can be related to family members, especially when adult children and grandchildren live in the receiving country, too (Albert et al. 2016a, b; Victor and Zubair 2016; Bolzman et al. 2006).

Further, transmitting values and norms is part of the family identity and might provide a sense of safety to immigrants in the new cultural context (Cigoli and Scabini 2006; Ferring 2017).

However, first-generation parents might consider intergenerational value continuity to be more important as compared to their offspring (see intergenerational stake hypothesis, Bengtson and Kuypers 1971). Immigrant parents originating from more collectivist and family-oriented cultural contexts might find it difficult to reconcile their expectations for social contact with the usual practices and habits in a more individualistically oriented receiving country (Burholt et al. 2018). This can lead to ambivalences and conflicts in parent–child relationships (Albert and Coimbra 2017).

With regard to intergenerational belonging it is hypothesised thus that family cohesion and perceived intergenerational value consensus will protect against perceived loneliness, whereas family conflict will increase perceived loneliness.

As previously mentioned, the family constitutes an important resource and a refuge for migrants particularly in times of difficulty. Numerous studies have shown a tendency for immigrants to focus strongly on their immediate family in the receiving country (Albert and Barros Coimbra 2017; Baykara-Krumme and Fokkema 2019; Burholt and Dobbs 2014; Genoni and Nauck 2018; Rooyackers et al. 2014). However, an ‘acculturation gap’ might evolve between first-generation parents who experience difficulties adjusting to the new living context and their second-generation children who adapt faster to the receiving context (see e.g. Birman 2006). In such a constellation, the migrants’ lack of cultural belonging to the receiving country could widen the gap to their children and also translate into difficulties in their intergenerational relations, which in turn could increase their feelings of loneliness.

Therefore, it is hypothesised that the impact of cultural belonging on perceived loneliness will be mediated by intergenerational belonging.

### Portuguese immigrants in Luxembourg

Foreign nations make up 47% of the population of Luxembourg with Portuguese immigrants constituting the largest part of this group. The number of Portuguese nationals currently living in Luxembourg is 95,057, representing about 15% of the total population (STATEC 2020). Corresponding to the increasing labour demands in Luxembourg’s industrial and service sectors, the migration of Portuguese nationals to Luxembourg began in the late 1960s. These first-generation migrants are now close to retirement age (see also Kühn 2015). Earlier findings showed that the majority of Portuguese first-generation migrants planned to stay in Luxembourg after retirement or to commute between countries, with only a smaller number wanting to return to Portugal (Albert et al. 2016a, b). This typical pattern has also been reported for other migrant groups across Europe (Albert et al. 2016b; Attias-Donfut et al. 2005).

### Research questions

The aim of the present study is to predict perceived loneliness from aspects of cultural and intergenerational belonging. I predict that feelings of cultural belonging and intergenerational belonging within the family can be protective factors against loneliness, whereas feelings of not fitting in culturally or within the intergenerational bonds of the family might be a risk factor for loneliness. Specifically, the following hypotheses were tested.

1. Cultural attachment to Luxembourg (LU) and Portugal (PT) as well as the feeling that both cultures are compatible and that one can competently navigate between the two will protect against perceived loneliness, whereas acculturative stress and the feeling that both cultures are conflicting will increase perceived loneliness.
2. Family cohesion and perceived intergenerational value consensus will protect against perceived loneliness, whereas family conflict will increase perceived loneliness.
3. The impact of cultural belonging on perceived loneliness will be mediated by intergenerational belonging.

## Methods

### Sample

The present study is part of the FNR-funded IRMA project (Intergenerational Relations in the Light of Migration and Ageing, PI: Dr. Isabelle Albert) on intergenerational family relations between ageing parents and their adult children in Luxembourgish families and Portuguese migrant families, all living in the Grand Duchy of Luxembourg. In the project, two family generations of Portuguese immigrants in Luxembourg—adult children and their parents—were compared to Luxembourgish families.

Only the data of the Portuguese parent generation were used in the current analyses. This subsample comprises *N* = 131 Portuguese participants^2^ with an average age of *M* = 56.08 (SD = 7.80; range: 41–80) years, slightly over half of whom were female (51.9%). All participants were born in Portugal and had come to Luxembourg at an average age of *M* = 24.26 (SD = 7.49) years. Most participants (90.6%) were married to a Portuguese partner, and all were parents of at least one adult child who grew up and is still living in the Grand Duchy of Luxembourg. The majority was or had been employed in the service and construction sectors and indicated an average socio-economic status. They had on average a rather low educational status. Detailed socio-demographic indicators are presented in Table 1. Overall, the socio-demographic characteristics of our sample reflect the typical profile of the first waves of Portuguese immigrants in Luxembourg (Heinz et al. 2013).

**Table 1:** Socio-demographic variables

Variable	M	SD	Range
Age of participants	56.08	7.80	41–80
Length of stay in LU	31.71	8.81	11–50
Average age at arrival to LU	24.26	7.49	10–48
Number of children	2.25	0.73	1–5
Household size (including participant)	3.35	1.22	1–7
Self-perceived health status	3.32	0.65	1–5
Socio-economic status	2.47	0.75	1–5

	%	*n*	
Gender (female)	51.9	68	
*Educational status*			
No school certificate	11.5	15	
Elementary school	60.8	79	
Secondary school	14.6	19	
Professional certificate	11.5	15	
Other	1.5	2	
Retired	36.0	45	
Married/living with partner	89.3	117	

### Measures

A standardised questionnaire, initially developed in German, was translated into French and Portuguese by a team of multilingual psychologists. This translation process followed all the standards required to construct equivalent questionnaire versions (such as employing an independent check of congruence of translations and original version as well as subsequent discussion of incongruence among multilingual experts until agreement). The existing translations of well-known instruments included in this questionnaire were reviewed and considered in decisions for final item wordings. About 76.3% of the Portuguese first-generation participants chose the Portuguese language version of the questionnaire; the remainder chose the French version.

#### Cultural belonging

To assess cultural belonging, three measures were used, namely attachment to LU and PT culture, cultural identity and acculturation stress.

Participants’ cultural attachment to both the host country as well as their country of origin was assessed by a newly developed scale containing pictures or icons of PT and LU national/cultural symbols (Marinho Ribeiro 2014; see also Barros and Albert 2019). Participants had to rate their attachment to the different symbols, which represented aspects such as food, athletes or the national flag (14 items in total, 7 for each culture) on a 5-point Likert scale ranging from 1 (*not attached at all*) to 5 (*very attached*). These symbols were selected to evoke participants’ cultural knowledge and emotions and are similar to those used by Hong et al. (2000). Reliabilities were *α* = 0.80 for PT attachment and *α* = 0.84 for LU attachment.

To assess cultural identity, we used a shortened and adapted version of the BIOScale (Bicultural Identity Orientation; Comănaru 2009; see also BII; Benet-Martinez and Haritatos, 2005) with 13 items (for details see Barros and Albert 2019) rated on a 6-point Likert scale from 1 (*do not agree at all*) to 6 (*totally agree*). With three items, the first dimension referred to a compatible identity orientation (*α* = 0.60; e.g. ‘I believe that my identity is a mixture of the Portuguese and the Luxembourgish culture’). This dimension is in line with a hybrid identity where people are aware of their double cultural belonging, experiencing both cultures as compatible and being committed to both of their cultures. The second dimension measured conflicted identity orientation with five items (*α* = 0.75; e.g. ‘Sometimes I feel I’m really confused about my cultural identity’). This dimension describes people’s difficulties to reconcile both of their cultures and their confusion as to where they belong. The last dimension consisted of three items (*α* = 0.74) and referred to the behavioural aspect of cultural frame switching (e.g. ‘I'm adapting my cultural identity to the respective context’). This dimension reflects participants’ acknowledgement that they alternate their behaviour depending on the situation and the people they are with, namely from the culture of origin or from the receiving culture.

Furthermore, employing a short version (7 items) of the Riverside Acculturation Stress Inventory (RASI; Benet-Martinez and Haritatos 2005), participants were asked to evaluate their acculturative stress caused by the migrant situation on 6-point Likert scales ranging from 1 (*do not agree at all*) to 6 (*fully agree*). This measure refers to multiple acculturative stressors such as language difficulty, stress related to interpersonal relations and experiences of discrimination (e.g. ‘I have been treated unkindly or unfairly because of my Portuguese origin’). The reliability of this measure was *α* = 0.75.

#### Intergenerational belonging

Three family variables were assessed to determine respondents’ intergenerational belonging: climate in the nuclear family with two subscales and intergenerational value consensus with one’s own children.

Two dimensions of family climate referring to the nuclear family (first-generation parents and their adult children) were assessed (see Manzi et al. 2006; Schneewind 1988). Four items each were used to measure (1) family cohesion (e.g. ‘In our family, there’s a strong feeling of togetherness’) by assessing the sense of mutual support in the family and being available for each other in case of need and (2) family conflict (e.g. ‘In our family, we are often angry at each other’) by referring to open conflicts, arguments, antagonism and anger between family members. All items were responded to on a 6-point Likert scale ranging from 1 (*do not agree at all*) to 6 (*fully agree*). Reliability was *α* = 0.63 for family cohesion and *α* = 0.74 for family conflict.

Intergenerational value consensus with own children was measured by three items (e.g. ‘How strongly do you think that your children have taken over the values that are important for you?’) which had been used in an earlier study (see Albert and Ferring 2012). This dimension referred to the parents’ perception of how similar their adult children’s values were to their own and how strongly they had influenced each other. Items were rated on a Likert scale ranging from 1 (*very weakly*) to 6 (*very strongly*); reliability of this scale was *α* = 0.80.

#### Perceived loneliness

Perceived loneliness was assessed using a short, 3-item version of the R-UCLA Loneliness Scale (Hughes et al. 2004; for the long version see Russell et al. 1980) that had to be rated on a 5-point Likert scale from 1 (*never*) to 5 (*always*). This scale was chosen for two reasons: The short version has been tested in older adult samples and has been widely used in large surveys, such as the English Longitudinal Study of Ageing (ELSA) and the Irish Longitudinal Study of Ageing (TILDA), and its application is recommended in studies where participants are asked to fill out questionnaires on their own (Campaign to End Loneliness 2020). The original scale contains 20 items pertaining both to feelings of loneliness and social isolation. The short scale focusses on relational and social connectedness as well as perceived isolation. More precisely, the items in the short scale asked participants to assess how often they felt that they lacked companionship, felt left out as well as felt isolated from others. With satisfactory reliabilities, the short version has proven useful, reaching a reliability of *α* = 0.76 in the present study.

For all measures, the mean was calculated and used for further analyses.

### Procedure

Participants were recruited via different interest groups, advertisements (flyers, radio and newspaper) and a lecture series, as well as word-of-mouth advertising. Participation in the study was voluntary, and participants provided informed consent. Each family member received 10 euros as a reward for participation. The Ethics Review Panel (ERP) of the University of Luxembourg approved the study.

## Results

To analyse the protective role of cultural and intergenerational family belonging against perceived loneliness, I first calculated correlations and subsequently conducted a hierarchical regression analysis to determine the predictive value of the different indicators. In the first step, the impact of socio-demographic variables was identified, in the second step, variables of cultural belonging were added, and in the last step, variables of intergenerational family belonging were added to the model. As I assumed that cultural belonging would refer to more distal aspects of a person’s surroundings and intergenerational family belonging would pertain to the immediate living context of a person, a mediator analysis by use of the PROCESS macro for SPSS (Hayes and Rockwood 2017) was lastly conducted to examine if the predictive value of cultural belonging for perceived loneliness would be reduced when taking into account intergenerational family belonging.

### Correlation analyses

With regard to the participants’ socio-demographic characteristics, as expected and in line with earlier studies, length of stay, socio-economic status as well as health status were all related to perceived loneliness (see Table 2). Older migrants felt lonelier when they lived fewer years in Luxembourg, and when they evaluated their socio-economic status lower compared to others living in the country and with lower self-rated health status. Age at the time of the study, however, was not related to perceived loneliness. Interestingly, a correlation analysis with a composite indicator for age upon arrival in Luxembourg (the difference between age at interview and years of stay) was related to perceived loneliness (*r* (123) = 0.29, *p* < 0.01), in the sense that the older participants were upon their arrival, the lonelier they felt. There were no gender differences (women: *M* = 2.41, SD = 0.80 vs. men: *M* = 2.24, SD = 0.77, *t* (128) = 1.23, n.s.).

**Table 2:** Correlations between perceived loneliness and all further variables

	1	2	3	4	5	6	7	8	9	10	11	12	13	14	15
1	1	− 0.04	− 0.29**	− 0.30**	− 0.29**	0.31**	− 0.18*	− 0.10	0.21*	− 0.04	− 0.07	0.33**	− 0.22*	0.50**	− 0.25**
2		1	0.59**	− 0.28**	− 0.05	0.32**	0.02	0.07	0.17	0.05	0.10	0.08	0.18*	− 0.17	0.14
3			1	0.08	0.02	− 0.58**	0.27**	0.05	0.19*	0.29**	0.28**	− 0.18	0.19*	− 0.26**	0.09
4				1	0.15	− 0.38**	0.11	− 0.06	− 0.06	0.18*	0.11	− 0.30**	0.08	− 0.02	0.09
5					1	− 0.05	0.07	− 0.08	− 0.01	0.01	0.15	− 0.18*	0.17	− 0.12	0.23**
6						1	− 0.26**	0.02	− 0.08	− 0.30**	− 0.25**	0.23**	− 0.03	0.09	0.01
7							1	0.33**	0.01	0.35**	0.27**	− 0.21**	0.08	− 0.25**	0.22**
8								1	0.04	0.16	0.11	0.26**	− 0.01	− 0.04	0.14
9									1	0.00	0.31**	0.27**	0.07	0.19*	0.06
10										1	0.35**	− 0.04	0.04	− 0.05	0.22*
11											1	− 0.03	0.02	0.04	0.05
12												1	− 0.11	0.23**	− 0.06
13													1	− 0.10	0.41**
14														1	− 0.23**
15															1

1 = Perceived Loneliness, 2 = Age of Participant, 3 = Length of Stay in LU, 4 = Socioeconomic Status, 5 = Health Status, 6 = Age at Arrival in LU, 7 = Cultural Attachment to LU, 8 = Cultural Attachment to PT, 9 = Cultural Identity Conflict, 10 = Compatible Cultural Identity, 11 = Cultural Frame Switching, 12 = Acculturative Stress, 13 = Family Cohesion, 14 = Family Conflict, 15 = Intergenerational Value Consensus

Findings for the variables pertaining to cultural belonging reveal that attachment to Luxembourg correlated significantly with lower perceived loneliness (*r* (129) = − 0.18, *p* < 0.05), whereas attachment to Portugal showed no correlative relation with perceived loneliness. With regard to cultural identity, only a conflicted identity was significantly and positively related to perceived loneliness (*r* (129) = 0.21, *p* < 0.05), whereas no significant relations were found between a compatible cultural identity or frame switching and felt loneliness. Finally, acculturation stress was strongly correlated with loneliness (*r* (129) = 0.33, *p* < 0.01). Concerning intergenerational family belonging, all assessed variables were significantly correlated with perceived loneliness. Value consensus (*r* (128) = − 0.25, *p* < 0.01) and family cohesion (*r* (130) = − 0.22, *p* < 0.05) were negatively correlated with perceived loneliness, and in contrast, family conflict (*r* (130) = 0.50, *p* < 0.01) was positively correlated.

### Regression analyses

In the regression analyses, only those aspects were included that were correlated significantly with perceived loneliness: socio-demographic aspects in the first step, cultural belonging in the second step and intergenerational belonging in the third step.

With regard to the participants’ socio-demographic characteristics, whereas length of stay, socio-economic status as well as health status predicted perceived loneliness in the first step, the effect of length of stay vanished in the last step when the intergenerational relationship variables were included.

With regard to cultural belonging, including cultural attachment to LU, cultural identity conflict and acculturative stress, a conflicted identity was the strongest and only predictor for loneliness (see Table 3).

**Table 3:** Regression model to predict perceived loneliness by socio-demographic variables, cultural belonging and family belonging (*N* = 131)

Variable	Model 1			Model 2			Model 3		
	*B*	SE* B*	β	*B*	SE* B*	β	*B*	SE* B*	β
Time in Luxembourg	− 0.019	0.009	− 0.205*	− 0.020	0.009	− 0.220*	− 0.007	0.008	− 0.075
Socio-economic status	− 0.229	0.093	− 0.213*	− 0.181	0.092	− 0.168	− 0.180	0.083	− 0.168*
Health status	− 0.299	0.097	− 0.247**	− 0.277	0.094	− 0.228**	− 0.196	0.087	− 0.162*
Age at arrival	0.010	0.011	0.091	0.008	0.011	0.077	0.017	0.010	0.156
Attachment to LU				− 0.044	0.078	− 0.045	0.044	0.073	0.045
Conflicted cultural identity				0.175	0.067	0.211*	0.119	0.062	0.143
Acculturation stress				0.086	0.063	0.118	0.050	0.057	0.068
Family cohesion							− 0.109	0.087	− 0.096
Family conflict							0.321	0.064	0.385**
Intergenerational value consensus							− 0.077	0.075	− 0.082
Δ *R*^*2*^	0.22**			0.07*			0.15**		
*F* (df) for change in *R*^*2*^	8.73** (4, 126)			4.18* (3, 123)			10.81** (3, 120)		

*p < 0.05; **p < 0.01 missing values were substituted by mean

Regarding variables of intergenerational belonging, namely intergenerational value consensus as well as family climate within the nuclear family (cohesion and conflict), only conflict was predictive in the regression model (see Table 3).

As predicted, the effect of cultural identity conflict was reduced to insignificance when introducing intergenerational family conflict in the third step. Thus, further testing examined the mediator effect of family conflict on the relation between conflicting cultural identity and perceived loneliness. As assumed, cultural identity conflict was a predictor of family conflict (*B* = 0.21, *SE B* = 0.09, *t* = 2.42, CI [0.0383; 0.3797]), and the indirect effect of cultural identity conflict on perceived loneliness via the mediator intergenerational family conflict was *B* = 0.08; SE* B* = 0.03, CI [0.02; 0.15].

## Discussion

In the present study, I set out to explain feelings of loneliness and social isolation of long-term Portuguese migrants in Luxembourg by looking at aspects of cultural and intergenerational belonging. I hypothesised that feelings of cultural and intergenerational belonging would be protective factors against loneliness, whereas the feeling of not fitting in might be a risk factor for loneliness and that the impact of cultural belonging on perceived loneliness would be mediated by intergenerational family belonging.

Importantly, the participants’ socio-demographic characteristics were highly significant in predicting loneliness. There are two interrelated aspects linked to time that are important for loneliness feelings: the duration of time one spent at the destination and the age at the time of migration. Absolute time spent in Luxembourg reduced felt loneliness, whereas age per se was not related to loneliness. This is in line with the assumption that, with time, new social contacts and bonds are built up in the host society (see Bolzman et al. 2006). Interestingly, the older the participants were at the time of migration to Luxembourg, the lonelier they felt. This is an important result as, apart from having spent less time in the receiving country, it underlines the significance of developmental stage at the time of migration. As Titzmann and Lee (2018) note, the adaptation in the context of migration occurs at different points in the life span, in parallel with specific further developmental tasks. It might be easier for younger people to adapt to a new living context compared to older people who have already lived longer in another country, which has left an ‘imprint’ and where they might already have established lasting social bonds (see the concept of proculturation, Gamsakhurdia 2018). Interestingly, length of stay was no longer significant when including intergenerational belonging—the intergenerational embeddedness might compensate for further cultural or social links in the receiving society.

Furthermore, health was significantly related to loneliness in that healthier people were less lonely, and socio-economic status also reduced loneliness, which is in line with earlier findings (De Gierveld and Van Tilburg 2010; Fokkema and Naderi 2013). In turn, health problems might impair mobility and cause difficulties in keeping social contacts alive, whereas economic difficulties can undermine participation in social encounters.

With regard to cultural belonging, I focussed on aspects of cultural attachment to LU and PT, cultural identity and acculturative stress. Whereas cultural attachment to Luxembourg reduced feelings of loneliness, cultural identity conflict and acculturative stress increased the feeling of loneliness. Cultural attachment included national symbols, pictures of typical places, foods and famous people; it referred thus to the familiarity of geographical places and symbolic bonds pertaining to feeling at home. This seems particularly important in reference to the receiving context where participants spend most of their current time. The ability to act efficaciously in this specific living context might be undermined if one feels discriminated against or when experiencing difficulties (e.g. related to language competences) as indicated by acculturative stress. In this vein, in the present study, the knowledge of some Luxembourgish was—in spite of generally rather low self-rated Luxembourgish language ability—beneficial and protective against the feeling of loneliness. The strongest predictor was, however, a conflicted cultural identity. These results indicate that although attachment to the cultural context one lives in might be a protective factor, the experience of difficulties in reconciling different cultural belongings seems to be the most important for feelings of loneliness in the context of migration. At the intergroup level, according to Bourhis et al. (1997), relations between immigrants and the receiving society can be harmonious, problematic or antagonistic, depending on the compatibility between acculturation orientations and expectations of both groups. Evidently, when intercultural relations are experienced as difficult, this can also have an effect on the individual level as our results indicate.

Effects of cultural embeddedness, however, vanished when intergenerational relations of our participants were taken into account as proximal living context. A family climate of mutual understanding, where family members are there for each other and share common values, reduced loneliness. Thus, it seems that the feeling of having a family network one can count on is of prime importance. Family cohesion refers to a strong feeling of togetherness and support which is a protective factor against loneliness. In the same vein, the experience of intergenerational value consensus with one’s own children reduced feelings of loneliness, thus providing a feeling of understanding, connectedness, continuity and family identity (Barni and Donato 2018; Barni et al. 2013; Cigoli and Scabini 2006). However, conflicted intergenerational relations were strongest in predicting loneliness. In fact, in the regression model considering all family variables, only family conflicts were predictive of greater loneliness. This is in line with Baumeister and Leary's (1995) suggestion that people have a high need for social contacts that are not conflict-laden. Family conflict is thus a strong risk factor for loneliness, as it might undermine the sense of belonging and togetherness. In addition, family conflict was a mediator of the relation between cultural identity conflict and perceived loneliness. Personal difficulties in reconciling the different ways of life in the culture of origin and the receiving culture seem to go hand in hand with intergenerational family conflicts in the context of migration, which lead in turn to feelings of loneliness. An acculturation gap between first- and second-generation family members can thus have far-reaching consequences for immigrants’ subjective well-being. This result underlines the immediate importance of the nuclear family as proximate living context and as a particular point of reference for first-generation migrants.

### Limitations

I focussed here on a carefully defined sample of adult first-generation, long-term Portuguese migrants that were living with their families in Luxembourg. The sample size was rather small, in part due to the specific study design that stipulated a participation precondition including the spouse as well as one adult child, making it more difficult to find a large number of participants. The importance of family that was found for these participants is possibly because they had all founded a family in Luxembourg, and this finding should be explored in other groups, such as migrants to Luxembourg who remained single or without children living there. Regarding the sample, we also have to note the large age range of participants (41–80 years) in the present study which might also entail different relationship dynamics with adult children.

One further interesting indicator is the intention to stay in Luxembourg after retirement, return to their country of origin or commute between the two. In fact, for Amit and Bar-Lev (2015), the sense of belonging includes three components, namely national identity, feeling at home as well as commitment to stay in the host country. More than half of our sample indicated that they planned to stay in Luxembourg permanently, whereas a smaller share intends to commute or to go back to Portugal after retirement. Although these subgroups did not differ in their loneliness, we found different strategies of self-regulation, with those who intended to return to Portugal seeming to be less committed to putting down roots in Luxembourg (see Albert et al. 2016a, b).

The use of the UCLA Loneliness Scale has been criticised in studies on older adults as it was originally developed for use with younger participants. However, the adaptation of the scale for the purpose of this study was deemed acceptable because it refers to aspects of social connectedness and social isolation, which are supposed to be closely linked to the sense of belonging. In addition, it has been frequently used in self-report questionnaires employed in large surveys such as ELSA and TILDA.

## Conclusions

The findings of the present study have added to earlier results on cultural and intergenerational belonging and their roles in feelings of loneliness of adult immigrants. These results confirmed the expectations that were formulated on the roles of cultural and intergenerational belonging as protective factors and the feeling of not fitting in as risk factor of perceived loneliness. In particular, the feeling of not fitting in both culturally and socially within the family was a significant risk factor for the experience of loneliness. According to Baumeister and Leary (1995), the need to belong is universal. Their definition considers two criteria: the individuals’ need for positive (i.e. not conflict-laden) social interactions and their need for an affective, reliable and caring bond with others. The present study results confirm the importance of a sense of community and belonging for migrants’ well-being whereby, in particular, the feeling of not fitting in culturally might translate into intergenerational conflicts within the family, which in turn can have an important impact on the feeling of loneliness.

## Footnotes

1 This excerpt stems from an interview conducted in the framework of the IRMA-study with a Portuguese mother (66) and her adult daughter (45) from a migrant family in Luxembourg. The family migrated to Luxembourg at an early age of the daughter; the mother had already made a further brief migration experience in a third country.

2 We excluded one participant from our original first-generation sample who had come to Luxembourg at the age of 7 as well as two participants who indicated that they live full-time in Portugal.
